# Supplementary material for: Venomix: a simple bioinformatic pipeline for identifying and characterizing toxin gene candidates from transcriptomic data
Source: PeerJ. 2018 Jul 31;6:e5361. doi: 10.7717/peerj.5361 (PMC6074769; doi:10.7717/peerj.5361)
Supplement: Supplemental Information 3 [file peerj-06-5361-s003.gz › FinalOutput_E-20/Snaclec_echicetin_subunit_beta_6/finaltree.pdf]

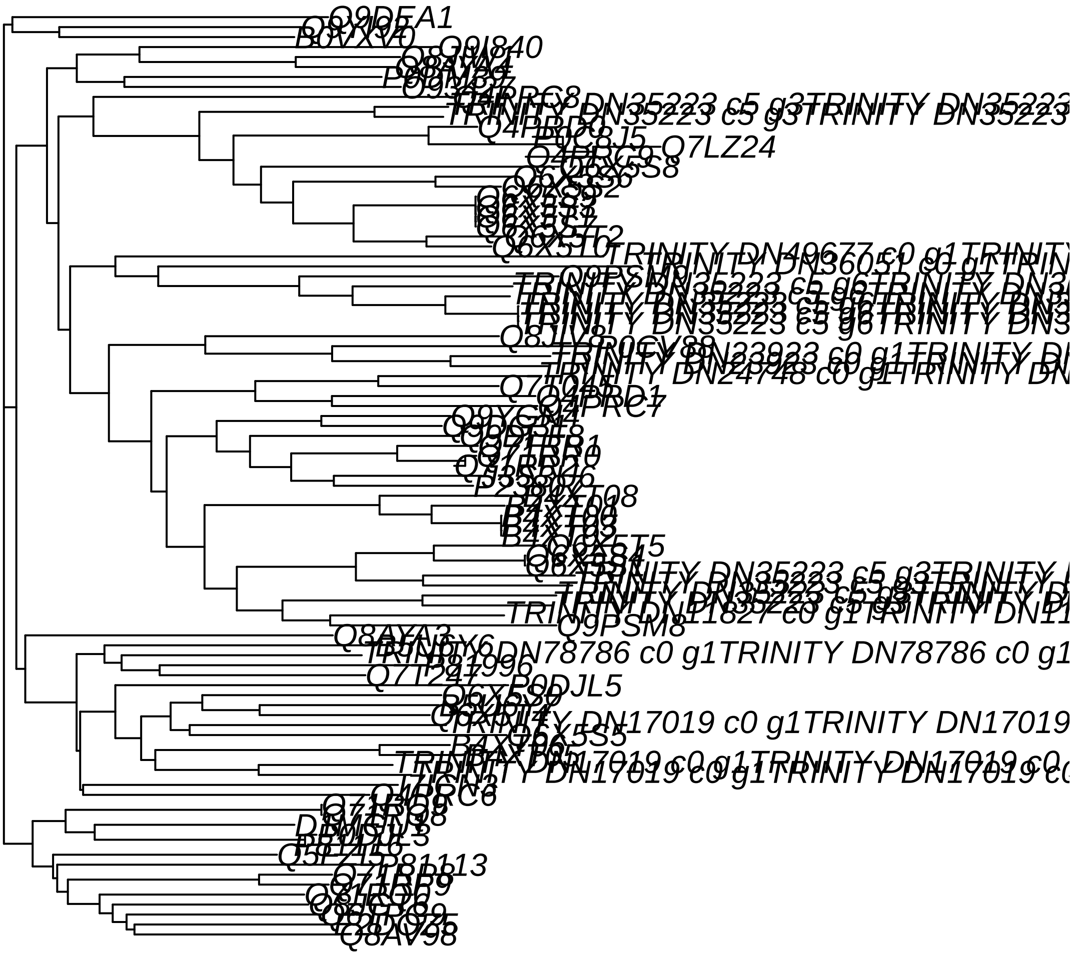
Q9DFA1
B0VXV0
Q9I840
Q7LZ24
Q6X5S8
Q8JIV8
Q7T045
Q4PRC7
Q8AYA3
TRINITY DN78786 c0 g1TRINITY DN78786 c0 g1
Q7T241
P0DJL5
Q6X5S5
Q9PSM8
Q4PRC6
Q5PZ15
P81113
Q8AV98
